# Development of a multiplex fluorescence immunological assay for the simultaneous detection of antibodies against *Cooperia oncophora*, *Dictyocaulus viviparus* and *Fasciola hepatica* in cattle

**DOI:** 10.1186/s13071-015-0924-0

**Published:** 2015-06-19

**Authors:** Sofia N. Karanikola, Jürgen Krücken, Sabrina Ramünke, Theo de Waal, Johan Höglund, Johannes Charlier, Corinna Weber, Elisabeth Müller, Slawomir J. Kowalczyk, Jaroslaw Kaba, Georg von Samson-Himmelstjerna, Janina Demeler

**Affiliations:** Institute for Parasitology and Tropical Veterinary Medicine, Freie Universität Berlin, Berlin, Germany; UCD School of Agriculture, Food Science and Veterinary Medicine, University College Dublin, Dublin, Ireland; Department of Biomedical Sciences and Veterinary Public Health, Section for Parasitology (SWEPAR), Swedish University of Agricultural Sciences, Uppsala, Sweden; Department of Virology, Parasitology and Immunology, Faculty of Veterinary Medicine, Ghent University, Ghent, Belgium; LABOKLIN GMBH & Co.KG, Bad Kissingen, Germany; Laboratory of Veterinary Epidemiology and Economics, Faculty of Veterinary Medicine, Warsaw University of Life Science, Warsaw, Poland

**Keywords:** Parasitic gastroenteritis, Lungworm, Liver fluke, Luminex, Multiplex immunoassays, Serum, Cattle, Diagnosis

## Abstract

**Background:**

A major constraint for the effective control and management of helminth parasites is the lack of rapid, high-throughput, routine diagnostic tests to assess the health status of individual animals and herds and to identify the parasite species responsible for these helminthoses. The capability of a multiplex platform for the simultaneous detection of three pasture associated parasite species was evaluated and compared to existing ELISAs.

**Methods:**

The recombinant antigens 14.2 kDa ES protein for *Cooperia oncophora*, major sperm protein for *Dictyocaulus viviparus* and Cathepsin L1 for *Fasciola hepatica* were recombinantly expressed either in *Escherichia coli* or *Pichia pastoris*. Antigens were covalently coupled onto magnetic beads. Optimal concentrations for coupling were determined following the examination of serum samples collected from experimentally mono-infected animals, before and after their infection with the target species. Absence of cross-reactivity was further determined with sera from calves mono-infected with *Haemonchus contortus, Ostertagia ostertagi* and *Trichostrongylus colubriformis*. Examination of negative serum samples was characterised by low median fluorescence intensity (MFI).

**Results:**

Establishment of the optimal serum dilution of 1:200 was achieved for all three bead sets. Receiver Operating Characteristic analyses were performed to obtain cut-off MFI values for each parasite separately. Sensitivity and specificity at the chosen cut-off values were close to, or 100 % for all bead sets. Examination of serum samples collected on different days post infection from different animals showed a high reproducibility of the assays. Serum samples were additionally examined with two already established ELISAs, an in-house ELISA using the recombinant MSP as an antigen and a DRG ELISA using Cathepsin L1 for liver fluke. The results between the assays were compared and kappa tests revealed an overall good agreement.

**Conclusions:**

A versatile bead-based assay using fluorescence detection (xMAP® technology) was developed to simultaneously detect antibodies against *C. oncophora, D. viviparus* and *F. hepatica* in cattle serum samples. This platform provides rapid, high-throughput results and is highly sensitive and specific in comparison to existing serological as well as coproscopical diagnostic techniques.

## Background

Nematode and trematode infections play an important role for animal welfare and are of great concern for the economy of the global ruminant livestock industry today [1]. Constantly increasing financial costs for anthelminthic prophylaxis and treatment due to the spread of anthelmintic resistant parasite populations, as well as the often overlooked subclinical effects of the helminth infections on animal productivity [2, 3] have led to the need of developing new and sustainable strategies concerning the effective control of helminthoses. An important step towards this end is the development of new, efficient and high-throughput diagnostic techniques. Despite the development of more sensitive coproscopical methods [4], they often target individual animals and are not suitable for high-throughput diagnosis. Serological methods established so far appear to lack specificity, in particular when non-recombinant antigens are used, and multiple tests have to be performed to detect mixed species infections.

Amongst the helminths responsible for pasture-borne parasitoses the liver fluke, lungworm as well as gastrointestinal (GI) nematodes are the most important for cattle in temperate climate regions. *Cooperia oncophora*, a parasite of the small intestine of cattle occurs worldwide with high prevalence rates [5, 6]. It is usually associated with *Ostertagia ostertagi*, parasitising the abomasum, and both contribute to the complex of parasitic gastroenteritis (PGE) [1]. While *O. ostertagi* is known to be more pathogenic, *C. oncophora* is generally considered as the dose-limiting species [7], particularly for the macrocyclic lactone (ML) anthelmintics. Infections with these GI nematodes as well as with the bovine lungworm *Dictyocaulus viviparus* cause considerable decrease in weight gain in calves during the first grazing [8] season, can be responsible for significant reduction in milk yield [9, 10] and impair animal welfare [11]. The economic importance of such parasitic diseases has been repeatedly demonstrated [12], especially where intensive grazing management is practiced, and cost-benefit evaluations have indicated that financial losses due to the presence of GI nematodes can be high [2, 13]. Monitoring data provided by the Dutch Animal Health Service for *D. viviparus* indicate that the incidences of parasitic bronchitis tended to increase in the Netherlands [14]. In the same study, economic losses of approximately 160 € per cow were calculated. The liver fluke *Fasciola hepatica* affects large and small ruminants. In cattle, fasciolosis can appear as a chronic and subclinical form and is worldwide considered as one of the most important parasitic diseases causing substantial economic losses, which are estimated to be 2000 million $ (USA) per year in agriculture [15, 16]. Additionally, this parasite has zoonotic potential and environmental contamination through infected animals can be important for human health [17].

Diagnosis of these parasites is commonly based on coproscopical detection methods such as sedimentation (liver fluke), flotation (GI nematodes) or baermannisation of larvae (lungworm). Since eggs excreted by most GI nematodes are morphologically indistinguishable, species identification can only be achieved following faecal culturing or using molecular techniques [18–20]. The generally high handling costs as well as the necessity to sample several animals led to the increased use of serological methods which can be used for herd health monitoring. Serological diagnosis of *F. hepatica* has been described in the literature using excretory/secretory (ES) products [21–23], a “f2” antigen (Fasciolosis Verification Test, IDEXX, Hoofddorp, the Netherlands) and a recombinant Cathepsin L1 antigen [22]. The same applies for *D. viviparus* where the detection of antibodies in serum or milk using ELISAs with either crude ES antigen [24–26] or recombinantly expressed major sperm protein (MSP) [27–29] has been described. For the detection of *C. oncophora*, ELISA using crude antigen [30], or a recombinantly expressed 14.2 kDa ES protein [31] were reported.

All these ELISA only target a single species and in order to cover the spectrum of pasture-borne helminthoses, multiple assays have to be conducted. Recent technical advances offer the advantage of multiplex assays, resulting in higher throughput, increased flexibility, reduced sample volume and lower costs [32–34]. A popular multiplex platform is the bead-based Luminex® xMAP® technology (Luminex Corp., Austin, TX). The basis are different polystyrene beads which are labelled with distinct ratios of two fluorescent dyes (red and near-infrared), leading to more than 100 sets of distinguishable beads, which are also referred to as microspheres in the literature. With each set, different analytes can be measured in parallel in a single assay [35, 36]. Various ligands can be covalently conjugated to the surface of these beads. If antigens are used as ligands, assays equivalent to ELISAs can be developed. Interactions of the target analytes with antibodies is detected using biotinylated secondary antibodies and streptavidin, labelled with the reporter fluorochrome phycoerythrin (SA-PE). Fluorescence detection in the Luminex xMAP liquid suspension array system is achieved by two-laser flow cytometry [37]. This technology is relatively widely used in the field of human medical diagnosis [38] but only few reports have been published in the field of veterinary diagnosis, particularly regarding serological assays [39–41].

The aim of this study was to develop a new, versatile diagnostic assay for the simultaneous detection of specific antibodies against *F. hepatica*, *D. viviparus* and *C. oncophora* in cattle serum samples. The performance of the Luminex® platform was evaluated through comparison with already established ELISAs using the same or different antigens.

## Methods

### Serum samples and antigens

The standardisation of the assay was achieved by using control sera obtained from parasite naïve animals before (negative control) and after experimental mono-infection with the target parasites *D. viviparus*, (100 larvae over 5 consecutive days) *C. oncophora* (30,000–40,000 larvae) and *F. hepatica* (500 metacercariae). For testing specificity as well as cross reactivity, sera from animals mono-infected with other important GI nematodes, *Haemonchus contortus*, *Trichostrongylus colubriformis* and *O. ostertagi* were used. All animal experiments were conducted in strict accordance with the respective local legislation and the European guideline for animal experiments (2010/63/EU). They were approved by a) the Landesamt für Gesundheit und Soziales, Berlin, Germany under the reference number L 0088/10, b) the Ethical Commission of the Faculty of Veterinary Medicine, Ghent University, Belgium under the reference number EC2009/086 and c) the Swedish Animal Ethics Committee under the permission C4/2.

Additionally serum samples collected in Denmark (*n* = 39), Switzerland (*n* = 76) and Poland (*n* = 367) were used. In Denmark and Switzerland, samples were taken from grazing young cattle on randomly selected farms. The sampling in Poland took place on farms previously identified for a cross-sectional survey using a two-stage sampling approach [42]. On a subset of those farms, 10–15 first season grazing cattle were randomly sampled.

The antigen used for the detection of *F. hepatica* was a recombinant 37 kDa Cathepsin L1-like protein [43] provided by ILDANA BIOTECH, UCD, Dublin. It is an active site [Cys^26^Gly] mutant expressed in the yeast *Pichia pastoris*. For the detection of antibodies against *D. viviparus* the recombinant 43 kDa MSP expressed as a glutathione-S-transferase (GST) fusion protein in *Escherichia coli* BL21 (DE3) cells as previously described by Gozdzik *et al*. [27] was used.

### Production of recombinant *C. oncophora* ES 14.2 antigen

The protein used for the detection of antibodies against *C. oncophora* was a 14.2 kDa ES protein described previously by Poot *et al*. [31]. A codon optimised (*E. coli*) version of the open reading frame (ORF) was synthesised *in vitro* (SynthesisGene®; China). The ORF was amplified using the forward primer (5′-CAC CAA TGA ATA TAC CGA TGC ACT GGC AAA ATG TAC-3′) and reverse primer (5′-TTA TTC CCA ATA CAG ACA CAG AAC TTT CAG TT-3′). PCR products were cloned into the pET151 TOPO expression vector (Life Technologies). A Rosetta gami® (Novagene) *E. coli* clone containing the pET151/CoES14.2 was cultured at 37 °C until OD_600nm_ reached 0.6. Synthesis of the ES14.2-V5-6 × His protein was induced with 0.5 mM isopropylthio-galactoside (IPTG) at 37 °C for 4 h. The recombinant ES14.2-V5-6 × His protein was purified from inclusion bodies using Protino® Ni-IDA columns (Macherey-Nagel, Germany) according to the manufacturer’s protocol. An additional wash step using a 50 mM concentration of imidazole and 2 % Tween20 was conducted before elution with 250 mM imidazole. Purity of the eluted protein was analysed on 12 % SDS–PAGE, stained with GelCode™ colloidal coomassie stain (ThermoFisher). Western blotting using an anti-V5 antibody (Life Technologies) was carried out to confirm that the target protein was obtained.

### Antigen coupling to fluorescent beads

In order to remove sodium azide or imidazole, *D. viviparus* and *C. oncophora* recombinant antigens were purified by gel filtration using Micro Bio-Spin 6 chromatography columns (Bio-Rad, Germany) according to the manufacturer’s protocol. Concentrations of all antigens were determined using the CB-X™ Assay (G-Biosciences, USA).

*D. viviparus, C. oncophora* and *F. hepatica* antigens were conjugated on the surface of carboxylated magnetic beads (Bio-Plex Pro™ Magnetic COOH Beads – 1.25 × 10^7^ beads/ml, Bio-Rad) using the fluorescence regions 026, 062 and 065, respectively. Coupling reactions were performed using the Amine Coupling Kit® (Bio-Rad), following a two-step carbodiimide reaction protocol provided by the manufacturer. The stock suspension of uncoupled beads was vortexed at high speed for 30 s followed by sonication for 15 s in order to disperse bead aggregates. A 100 μl aliquot of monodisperse COOH beads (1.25 × 10^6^) was transferred to one Bio-Plex coupling reaction tube and was placed into the magnetic separator for 30 to 60 s before removal of the supernatant. The beads were washed once in 100 μl bead wash buffer, followed by re-suspension in 80 μl bead activation buffer. Then 10 μl of 50 mg/ml N-Hydroxysulfosuccinimide sodium salt (S-NHS) (Sigma-Aldrich, Germany) and 10 μl of 50 mg/ml N-(3-Dimethylaminopropyl)-N’-ethylcarbodiimide hydrochloride (EDAC) (Sigma-Aldrich), which were prepared in bead activation buffer immediately prior to their use, were added. The reaction tube was mixed gently, covered with aluminium foil and then gently agitated on a shaker for 20 min at room temperature (RT). PBS (pH 7.4, 150 μl) was added twice, always followed by vigorous vortexing. The recombinant protein was added and total volume was brought to 500 μl with PBS (final antigen concentration 5–12 μg/500 μl). Incubation was performed on a shaker at high speed (600–700 rpm) at RT for 2 h. In order to achieve higher coupling yields, some alterations of the initial protocol were made. It was observed that beads incubated at medium speed (500 rpm) as recommended had a tendency to precipitate at the bottom of the coupling reaction tube. Therefore, speed was slightly increased and the coupling reaction tube was vortexed once at high speed after 1 h to prevent precipitation.

Initially, different concentrations were used for each of the three antigens and were separately tested in order to determine the optimum antigen concentration. The amounts of the conjugated protein were the following for each bead-set: for *C. oncophora* 7 μg, 5 μg, 3.5 μg, 2.5 μg, 1.75 μg, 1.25 μg, 0.9 μg and 0.45 μg, for *D. viviparus* 5 μg, 2.5 μg, 1.25 μg, 0.66 μg and 0.45 μg, and for *F. hepatica* 5 μg, 2.5 μg and 1.25 μg. The coupled beads were placed into a magnetic separator for 1 min and after removal of the supernatant they were washed with 500 μl of PBS. The coupled beads were then re-suspended in 250 μl of blocking buffer and gently agitated at RT in the darkness for 30 min. Finally, the beads were washed with 500 μl of storage buffer, re-suspended in 150 μl storage buffer and stored at 4 °C in the dark. Beads were stored on the recommended conditions and always used within 4 months after coupling since decreased performance was observed thereafter.

### Luminex multiplex assay

The assays were conducted in 96-well polystyrene, round-bottom microplates (Greiner Bio-One). The three bead-sets were initially tested in singleplex assays including negative controls as well as the respective positive control sera. These were used in a two-fold eight serial dilution series in PBS/Tween20 (0.05 %, pH 7.4) in order to identify the optimal sample dilution. Cross-reactivity was assessed by running the assay with sera from calves infected with non-target species. Then, the three bead-sets were combined in a bead-mix and a multiplex assay was performed.

Prior to each examination, beads were re-suspended by vortexing and sonication for approximately 20 s three times to avoid high numbers of aggregated beads. A 50 μl aliquot of the working beadmixture (concentration 100 beads/μl) was transferred into the wells, followed by the addition of 50 μl of diluted sera. The plate was incubated on a plate shaker (800 rpm) in the dark at RT for 60 min. The plate was then placed into the magnetic separator and left for separation for 60 s. The supernatant was carefully removed from each well by manual inversion. Beads were washed 5 times by adding 100 μl PBS/Tween20 into each well to ensure absence of any undesirable or non-specifically bound antibodies. The plate was then removed from the magnetic separator and 100 μl of a biotinylated secondary antibody (goat anti-bovine IgG, Dianova, Germany) diluted 1:1000 in PBS/Tween20 were added to each well. Incubation was again conducted in darkness and at RT on a plate shaker (800 rpm) for 30 min before beads were washed as described above. Finally, 100 μl of streptavidin-phycoerythrin (SA-PE, Millipore) at 2 μg/ml, diluted in assay buffer, were added to each well. The plate was placed on the shaker, covered with aluminium foil and again incubated at RT on a plate shaker (800 rpm) for 30 min. The supernatant was carefully removed after magnetic separation of the beads by manual inversion and washing was performed as previously described. Assay buffer (100 μl) was added into each well and the plate was placed onto a plate shaker for approximately 15 s in order to achieve gentle agitation of the beads.

The beads were analysed using the Bio-Plex 200 instrument following the manufacturer’s instructions. A minimum of 100 events (beads) per well was read for every bead-set. All samples were analysed in duplicates in each run. To investigate reproducibility of the assays, several (between three and six) runs were performed using the sera from the same animals.

### ELISA used for comparison

Result obtained using the Luminex® assay were compared to existing ELISAs. *D. viviparus* antibodies were detected using either an in-house ELISA for lungworm based on the recombinant MSP antigen as described in von Holtum *et al*. [29] or using the modification of this ELISA as described by Gozdzik *et al*. [27]. Antibodies against *F. hepatica* were detected using either an in-house ELISA for liver fluke based on crude ES antigen following the method described by Salimi-Bejestani *et al*. [23] or the commercially available DRG liver fluke ELISAs (using recombinant Cathepsin L1). For the detection of *C. oncophora* antibodies no commercial ELISA is currently available and therefore no comparison was conducted.

### Statistical analysis

GraphPad Prism® software 5.04 was used for the statistical analyses. Five parameter logistic regression curves were calculated in order to determine the optimal serum dilution for each bead set separately. For differentiation, positive and negative samples as well as sera from animals infected with non-target species were compared using box plots. Negative and positive cut-off MFI values for each parasite specific assay were obtained using receiver operating characteristics (ROC) analysis to determine at the same time sensitivity and specificity. The respective values are automatically provided with the 95 % confidence intervals in the software used.

For comparison with existing ELISA (*F. hepatica* and *D. viviparus*) two subsets of 39 and 370 serum samples, respectively, were examined and Kappa tests were performed.

Percentages with confidence intervals and differences between countries were calculated using a Mid-P exact test in OpenEpi (http://www.openepi.com/Menu/OE_Menu.htm).

## Results

### Optimal amount of protein for coupling

The optimal amount of antigen identified for the target species were 0.45 μg for both, *D. viviparus* and *C. oncophora,* and 2.5 μg for *F. hepatica*. These were determined based on the amount of conjugated protein that would provide a reliable and reproducible MFI signal, which enabled a clear differentiation between positive and negative serum control samples.

### Optimisation of secondary antibody dilutions and SA-PE concentration

Using the optimal amount of antigen, four different secondary antibody dilutions (1:500, 1:1000, 1:2000 and 1:5000) and four SA-PE concentrations (0.5 μg/ml, 1 μg/ml, 2 μg/ml and 4 μg/ml) were tested. Optimal results were obtained for a 1:1000 dilution for the biotinylated secondary antibody and a concentration of 2 μg/ml for SA-PE.

### Optimal serum dilution

**Fig. 1:**
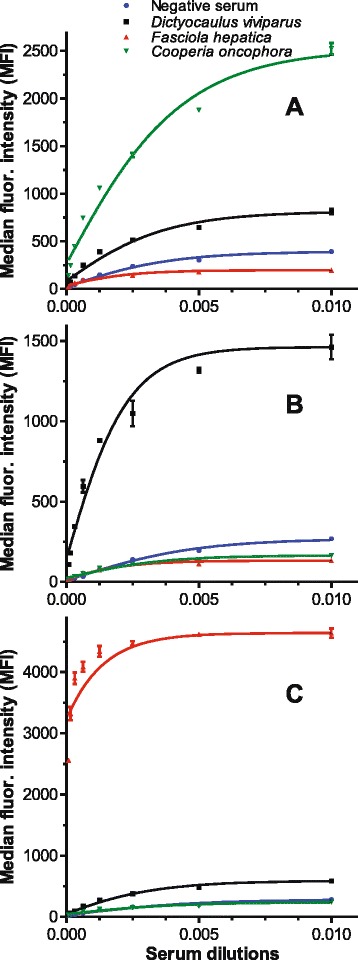
Results for singleplex assays using serum dilutions of cattle infected with *Dictyocaulus*, *Fasciola* and *Cooperia*. Five parameter logistic regression curves were calculated based on median fluorescence intensity (MFI) values. **a** *Cooperia oncophora* coupled beads using sera positive for *C. oncophora* (*green*), *Dictyocaulus viviparus* (black), *Fasciola hepatica* (*red*) and negative control sera (*blue*). **b** *D. viviparus* coupled beads and **c** *F. hepatica* coupled beads using the same sera. Dilutions are presented as 0.005 ≙ 1:200

**Fig. 2:**
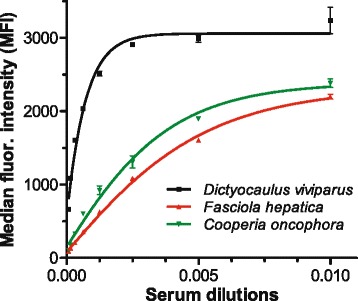
Results for the triplex assay using serum dilutions of cattle infected with *Dictyocaulus*, *Fasciola* and *Cooperia*. Five parameter logistic regression curves were calculated based on median fluorescence intensity (MFI) values. Beads are coupled with recombinant antigen for the detection of *Cooperia oncophora* (*green*), *Dictyocaulus viviparus* (*black*) and *Fasciola hepatica* (*red*). Artificial mixtures of sera from animals infected with the target species was used. Dilutions are presented as 0.005 ≙ 1:200

Determination of the optimal serum dilution was based on the examination of negative and positive control sera in a two-fold dilution series ranging from 1:100 to 1:12,800 for the separate coupled beadsets. The logistic regression curves of the MFI values for all three target species enabled a clear differentiation between the target species and negative control as well as non-target species (Fig. 1) with relatively low background MFI values. Multiplex assays were also performed using an 82-fold dilution series and results were comparable to those obtained in the singleplex assays (Fig. 2).

For all assays R^2^ values were close to 1. To achieve an optimal discrimination between positive and negative sera for all three bead sets a dilution of 1:200 was chosen.

### Assessment of cross-reactivity and cut-off determination

**Fig. 3:**
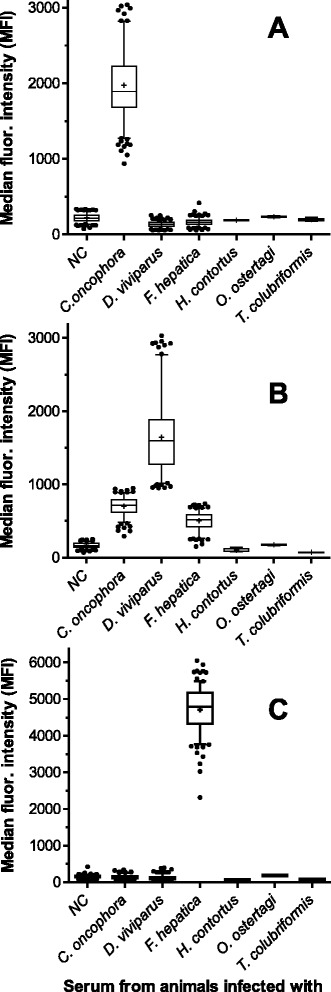
Cross reactivity analysis using sera from target and non-target species. Results are presented as box-plots showing the median fluorescence intensity (MFI) values obtained from multiple testing of sera from negative and mono-infected animals. Bead set were coupled with recombinant antigen for the detection of *Cooperia oncophora* (**a**), *Dictyocaulus viviparus* (**b**) and *Fasciola hepatica* (**c**). Whiskers represent 5 % and 95 % percentage quantiles and the mean is indicated by a +. Outliers are shown as individual dots

All bead sets were examined using positive and negative control sera as well as sera from non-target species infections. Since no obvious differences were observed between singleplex and multiplex assays, results were combined and are shown in Fig. 3. Box plots indicated clear differentiation between positive and negative control serum samples for all three target species. Regarding cross-reactivity antigens used for the detection of *C. oncophora* and *F. hepatica* could clearly distinguish between infections with target and non-target species (*H. contortus*, *T. colubriformis*, *O. ostertagi*, *D. viviparus* and *F. hepatica* or *C. oncophora*, respectively). This was different for the recombinant MSP antigen, where cross-reactivity was more pronounced for sera from *C. oncophora* and *F. hepatica* infected animals; particularly for a few *C. oncophora* positive sera differences to the lowest observed MFI value for *D. viviparus* were only minimal. Determination of the cut-off MFI values, sensitivity and specificity was achieved by ROC analysis separately for each bead set. Since serum samples were derived from experimentally infected animals and either clearly negative (parasite naïve prior to infection) or positive, two cut-off values were defined, one discriminating negative and one positive, leaving grey zone in between. For *D. viviparus* the situation was slightly different with some cross-reactivity present particularly for the *C. oncophora* coupled beads, so that for this assay only one cut-off value was determined. The cut-off values with sensitivity and specificity including the 95 % confidence intervals are presented in Table 1.

**Table 1:** Results of the Receiver Operating Characteristics analysis for negative (neg.) and positive (pos.) cut-off values. Median fluorescence intensity (MFI), sensitivity and specificity with confidence intervals (CI) are shown

	MFI	Specificity	95 % CI	Sensitivity	95 % CI
*C. oncophora*					
Neg. cut-off	379	99.84 %	99.11–100 %	100 %	98.17–100 %
Pos. cut-off	997	100 %	99.41–100 %	99.50 %	97.25–99.99 %
*D. viviparus*					
Cut-off	950.5	100 %	99.32–100 %	100 %	97.93–100 %
*F. hepatica*					
Neg. cut-off	340.8	99.46 %	97.01–99.99 %	100 %	98.02–100 %
Pos. cut-off	2670	100 %	98.02–100 %	99.46 %	97.01–99.99 %

### Assay reproducibility

Serum samples were obtained from different animals on different days pre and post infection and tested multiple times independently as well as in parallel on the same plate in order to determine the reproducibility of the assay. The MFI values obtained indicated reproducible results, which are shown in Table 2. The results obtained for the individual animals showed distinct immune responses, resulting in different levels of mean MFI values. Although CV were relatively high for some individuals when testing for antibodies against *D. viviparus*, all individual values clearly identified the respective samples as positive.

**Table 2:** Results of technical reproducibility using serum from different experimentally infected animals

	Animal 1	Animal 2	Animal 3	Animal 4	Animal 5
*C. oncophora*					
N	8	8	8	8	8
Mean MFI value	2236	3778	3870	3124	2876
CV	3.29 %	2.11 %	3.33 %	4.78 %	6.12 %
*D. viviparus*					
N	12	8	6	6	6
Mean MFI value	2332	3738	2595	1923	1472
CV	4.47 %	5.32 %	17.24 %	24.88 %	9.58 %
*F. hepatica*					
N	12	10	8		
Mean MFI value	1583	1888	4770		
CV	9.39 %	9.64 %	3.09 %		

*N* number of replicates

*MFI* median fluorescence intensity

*CV* coefficient of variation

### Comparison between multiplex assay and single ELISAs and field validation

The validation of this multiplex assay was performed by comparing the results obtained from the examination of individual serum samples with already existing and established assays.

Initially, only positive and negative samples derived from experimentally infected animals pre and post infection (*F. hepatica* and *D. viviparus*) were compared, using an in-house ELISA for lungworm as described by von Holtum *et al*. [29] as well as an in-house ELISA for liver fluke based on crude ES antigen. The results obtained were identical for both, negative as well as positive control sera.

To increase the number of samples tested, additionally serum samples collected during a field trial in Poland were used for comparison. Thirty-nine of these samples were analysed using the commercially available DRG liver fluke ELISAs. For the detection of lungworm no commercial ELISA is currently available. Serum samples were analysed in parallel in Sweden using an in-house ELISA [27]. In the latter, all samples were negative for *D. viviparus* antibodies while one sample was detected positive in the Luminex assay. Due to the fact that almost all samples were negative, no kappa statistic could be calculated. The comparison with the DRG liver fluke ELISA resulted in a kappa value of 0.37 (62.7 % of 0.60 maximum achievable). For this calculation the negative cut-off values for both assays were used. A larger subset of 363 samples were analysed using the commercially available SVANOVIR®F.hepatica-AbELISA (protocol identical to the in-house liver fluke ELISA as mentioned above) in the laboratory in Ghent. The comparison resulted in a kappa value of 0.460 (74.4 % of 0.67 maximal achievable). While 67 samples were positive only in the Luminex, there were also 21 samples which appeared positive in the ELISA but clearly negative in the Luminex assay.

Finally the newly developed triplex Luminex assay was used to analyse field serum samples collected in Denmark (n = 39), Switzerland (n = 76) and Poland (n = 367). The results obtained show no or low rates of lungworm infection in all three countries. *C. oncophora* appears in higher rates in Poland (73.8 %) in comparison to Denmark (28.2 %) and Switzerland (48.7 %). Additionally in Poland, increased levels of liver fluke were detected. Significant is the number of samples classified in the grey zone, which is relatively high in all three sampled countries. The calculated percentages are presented in Table 3.

**Table 3:** Percentage of serum samples positive for antibodies against *Cooperia oncophora*, *Dictyocaulus viviparus* and *Fasciola hepatica.* Results for the field samples are shown per country and include 95 % confidence intervals (95 % CI)

	Denmark (n = 39)	Poland (n = 367)	Switzerland (n = 76)
*C. oncophora*	28.21%^a^	73.84%^b^	48.68%^c^
95 % CI	16.42–43.90 %	69.11–78.08 %	37.78–59.71 %
*D. viviparus*	0%^ab^	3.82%^b^	0%^a^
95 % CI	0–10.68 %	2.23–6.34 %	0–5.77 %
*F. hepatica*	64.10%^ac^	79.84%^b^	67.11%^c^
95 % CI	48.73–77.31 %	75.42–83.63 %	55.91–76.65 %

Percentages which do not share the same indices (^a, b, c^) are significantly different in a Mid-P exact test (*p* < 0.05)

## Discussion

Pasture-borne parasitoses are highly prevalent in all grazing ruminants and have been recognised as important issues for animal welfare and productivity [2, 44]. This is particularly referring to the liver fluke, the lungworm and GI nematodes. Accelerated problems regarding anthelminthic resistance, climate change, intensification of farming and altered management practices have increased the need of development of new techniques in order to accurately diagnose and monitor these diseases.

In the current study, the successful development of a triplex assay for the simultaneous detection of antibodies against *C. oncophora*, *D. viviparus* and *F. hepatica* in serum from cattle is reported. While widely used singleplex assays using recombinant antigens were available for the latter two, expression of a *C. oncophora* antigen is described that was previously only once described for use in an ELISA. The new triplex assay was established using control sera from parasite naïve or controlled experimentally infected animals. Cross reactivity was assessed for all three antigens using the target parasites and revealed reliable detection with high sensitivity and specificity. Additionally, three non-target trichostrongylid nematode species (*H. contortus*, *O. ostertagi* and *T. colubriformis*) have been included in cross reactivity testing. Inclusion of *Nematodirus*, *Paramphistomum* and other important pasture-borne parasites would have been desirable, however, infection doses or serum of experimentally mono-infected animals were presently not available. Nevertheless, the evaluation of cross-reactivity presented here includes more parasite species than evaluated for many already commercialised assays.

Standardisation of any serological assay requires the use of defined negative and positive control sera collected from parasite naïve animals before and after specific mono-infection with the target parasites as well as non-target species. However, particularly regarding cross-reactivity, this information is often not accessible for commercially available assays. Also for the large number of developed and evaluated in-house ELISAs assessment of cross reactivity has often not been performed or at least not been reported. The specificity of the newly developed Luminex® assay was evaluated by testing the bead sets with sera obtained from animals infected with other important species for livestock (*H. contortus*, *O. ostertagi* and *T. colubriformis*). No cross-reactivity was obtained for all non-target species and all bead sets. For *C. oncophora* and *F. hepatica* also no cross-reactivity was detected for the other target species. For the detection of *C. oncophora* the 14.2 kDa recombinant antigen as reported by Poot *et al*. [31] was used. These authors reported no cross reactivity when serum samples from animals mono-infected with *O. ostertagi* or *D. viviparus* were used in their ELISA. This mirrors the result obtained in the current study, where no cross reactions were observed for any of the parasite species tested, leading to a sensitivity and specificity of 100 %.

For the detection of *F. hepatica* the recombinant Cathepsin L1 antigen was used. No cross reactivity was observed for any of the other species tested, also resulting a 100 % sensitivity and 100 % specificity. This is different to what has been reported in the literature regarding the use of this antigen. Kuerpick and colleagues [22] observed two false positive results out of 13 animals infected with *D. viviparus* and one out of four animals infected with *C. oncophora* (sensitivity between 90–100 %, specificity 88.6 %). Cornelissen *et al*. [45] obtained similar findings with five out of 191 animals infected with *D. viviparus*, one out of 31 animals infected with *C. oncophora* and one out of 55 animals infected with *O. ostertagi* (sensitivity 99.1 %, specificity 98.5 %). The currently observed absence of cross reactivity might not be confirmed in the Luminex® assay upon the use of significantly higher number of animals infected with non-target species. However, a similar level of cross reactivity as described above in the ELISAs would still be an improvement in comparison to systems using complex antigens such as ES antigen with reported specificities between 83–96 % [44].

The *D. viviparus* bead set was coupled with the recombinant MSP antigen. Although no or only minimal cross reactivity have been reported by von Holtum *et al*. [29] (sensitivity and specificity >99 %) as well as Gozdzik *et al*. [27] (sensitivity 97.7 %, specificity 98.1 %) for ELISAs using the same recombinant antigen, a few MFI values obtained for sera from animals infected with *C. oncophora* were very close to the lowest values of *D. viviparus* infected animals. However, no overlaps of MFI values occurred. In comparison to sera from negative animals or animals infected with *O. ostertagi* or *H. contortus*, MFI values obtained for sera from animals infected with *F. hepatica* and *C. oncophora* were substantially elevated. This background prevented the definition of two cut-off values separating clearly negative and clearly positive from the intermediate grey zone. The absence of two cut-off values for *D. viviparus* might complicate interpretation of field data in future epidemiological studies. In the field situation, some animals will be in the prepatency period or have already cured the infection but still have elevated antibody titres. MFI values for such animals can be expected to fall in the intermediate zone between negative and positive cut-off. Without such an intermediate zone, it will become difficult to identify such animals. Regarding *D. viviparus* it is also important to keep in mind that vaccination with attenuated larvae is possible. A cross reactivity of serum from vaccinated cattle is not expected since MSP is not expressed in premature stages and adult parasites do usually not develop. Additionally a sub-unit vaccine showing partial protection has been described recently [46]. Here cross reactivity should also not occur since entirely different recombinant antigens are used. However, experimental evidence for this is not available yet.

Out of the currently available coproscopical, serological and molecular techniques for the diagnosis and the identification of parasites only the first two are routinely used. The sensitivity and specificity of the different techniques are extremely variable. Serological assays have the advantage of their implementation as herd health monitoring tools. All currently available assays are only capable of diagnosing antibodies against one parasite and are often limited in their specificity due to the use of complex crude or ES antigens. Despite the fact that for all three parasite species of interest, *C. oncophora*, *D. viviparus* and *F. hepatica*, ELISAs using recombinant antigens have been described, only for the latter is a commercial ELISA using recombinant antigen available. Multiplexing diagnostic systems are well established in human medicine, but in veterinary medicine this technology has so far not been extensively used. However, during recent years several commercial or “in-house” bead-based multiplex assays have been developed, particularly in the field of virology [39, 47, 48], where existing ELISAs were compared with the new multiplexing assays revealing generally better performance for the latter. Additionally, xMAP technology has been used for the detection of inflammatory markers, [49–52]. In the field of parasitology, serological multiplex assays have only been reported for the simultaneous detection of *Trichinella spiralis* and *Toxoplasma gondii* in pigs [40, 41] and for the detection of *Plasmodium falciparum* in humans [53]. The Luminex® technology offers the advantage of reduced volume needed for diagnosis [39]. When multi-plexing assays, the costs per analyte are considerably lower in the Luminex® assay than in a standard ELISA, whereas the time needed for the examination of the samples is generally similar but reduced labour time per assay is achieved.

After the establishment of the Luminex assay, field samples obtained from naturally infected grazing animals during a trial in Poland have been used for comparison of the new Luminex® assay with already established ELISAs. In the case of multiplex bead-based immunoassays, comparison of the Luminex is frequently made with serological assays, such as ELISA. Correlation of these two methods varies among literature [34, 54–59]. In the current study, agreement between the assays was generally good when the same (recombinant) antigen was used. In case of the lungworm assay it was almost impossible to calculate kappa values since only one animal out of the 39 samples tested appeared positive (only in the new assay). The comparison of the DRG ELISA with Luminex was complicated by the fact that this ELISA uses five different categories and it was unclear, how to handle samples negative or positive in the Luminex assay and “questionable” or “low to moderate infection” in the other. Most of the deviations in classification were due to the fact, that the number of positive samples was higher in the Luminex assay but there were also one sample classified as negative in the Luminex but positive with the ELISA. Comparison of the Luminex® data with the ELISA using the ES antigen for *F. hepatica* detection had the advantage of a high number of samples tested. This resulted in a better kappa value than that obtained for the first comparison, however, there was a high number of samples positive in only one of the assays while being clearly negative (ES ELISA) or in the grey zone (Luminex®) in the respective other one. Since a recombinant antigen was used in the Luminex® assay a higher specificity can be expected than using a complex ES antigen. At the same time, this might result in decreased sensitivity since not all animals might develop antibodies against Cathepsin L1. However, more samples were detected positive with the Luminex® assay (67 vs 21), so sensitivity should not be of great concern here. Another explanation for the data obtained might be false positive samples in the ES ELISA, for example due to infection with closely related pathogens.

## Conclusion

A multiplex fluorescence serum immunoassay was successfully developed for the simultaneous detection of antibodies produced against *C. oncophora*, *D. viviparus* and *F. hepatica* in serum samples from cattle. It is characterised by low cost and time, high reproducibility and more importantly, by high sensitivity and specificity due to the use of recombinant antigens. This platform provides rapid, high-throughput diagnostic results, allowing the incorporation of further analytes and parameters in the future, such as hormones, inflammatory biomarkers and additional parasites. In particular, inclusion of *O. ostertagi* should be a major aim for the future, though recombinant antigens suitable for diagnosis are currently not available.
